# Metachronous Bilateral Adrenal Adenomas Causing Adrenocorticotropic Hormone-Independent Cushing’s Syndrome: A Case Report

**DOI:** 10.7759/cureus.103162

**Published:** 2026-02-07

**Authors:** I-Ting Hsiao, Chieh-Hua Lu

**Affiliations:** 1 Division of Pulmonary and Critical Care Medicine, Department of Internal Medicine, Tri-Service General Hospital, National Defense Medical University, Taipei, TWN; 2 Division of Endocrinology and Metabolism, Department of Internal Medicine, Tri-Service General Hospital, National Defense Medical University, Taipei, TWN

**Keywords:** adrenal cortical adenoma, adrenalectomy, bilateral adrenal cortical adenoma, cushing's syndrome, metachronous

## Abstract

Bilateral adrenal adenomas (BAAs) represent an uncommon etiology of adrenocorticotropic hormone (ACTH)-independent Cushing’s syndrome (CS); however, metachronous BAAs, where the adenomas present years apart, are exceptionally rare, with only a few cases previously reported. We present the case of a 53-year-old woman who developed ACTH-independent CS from a left adrenal cortical adenoma 19 years ago, treated successfully with a left-sided adrenalectomy. Nineteen years after the first episode, she presented with classic hypercortisolism symptoms, including central obesity, striae, and osteoporosis. Workup confirmed recurrent ACTH-independent CS with a low baseline ACTH level and a failure of both low- and high-dose dexamethasone suppression tests to suppress cortisol. Laparoscopic-assisted right-sided adrenalectomy was performed after image confirmation. Adrenal cortical adenoma was diagnosed by histological examination. Following the adrenalectomy, the patient required permanent glucocorticoid and mineralocorticoid replacement therapy. Serial brain magnetic resonance imaging (MRI) scans were initiated for Nelson's syndrome (NS) surveillance. Over 38 months of follow-up, the patient’s clinical symptoms improved, and no signs of NS were noted. This case represents the longest detailed reported case with an interval of 19 years between the diagnosis and surgical treatment of metachronous BAAs causing ACTH-independent CS. This rarity highlights the critical importance of long-term and regular follow-up for patients with unilateral adrenal adenoma to monitor for subsequent contralateral masses.

## Introduction

Cushing’s syndrome (CS) is a condition resulting from chronic exposure to excess glucocorticoids from any cause, most commonly long-term, high-dose use of cortisol-like medications. The disorder can also be caused by endogenous glucocorticoids, divided into adrenocorticotropic hormone (ACTH)-dependent (e.g., pituitary adenoma, ectopic ACTH secretion) and ACTH-independent (e.g., adrenocortical adenoma, carcinoma (ACC), nodular hyperplasia) [1].

Most CS are ACTH-dependent, up to 80-85% [2]. Among ACTH-independent CS, adrenal adenoma is the most common cause [3,4]. Within these cases, about 10-15% were caused by bilateral adrenal adenoma (BAA), most of them were diagnosed synchronously [5]. Metachronous BAA, causing ACTH-independent CS without other co-secretion, is extremely rare; only five cases have been reported to date [6-9]. Here, we report a rare case of metachronous ACTH-independent CS from left and right adrenal adenomas 19 years apart, the longest period that has been reported in detail.

## Case presentation

**Figure 1:**
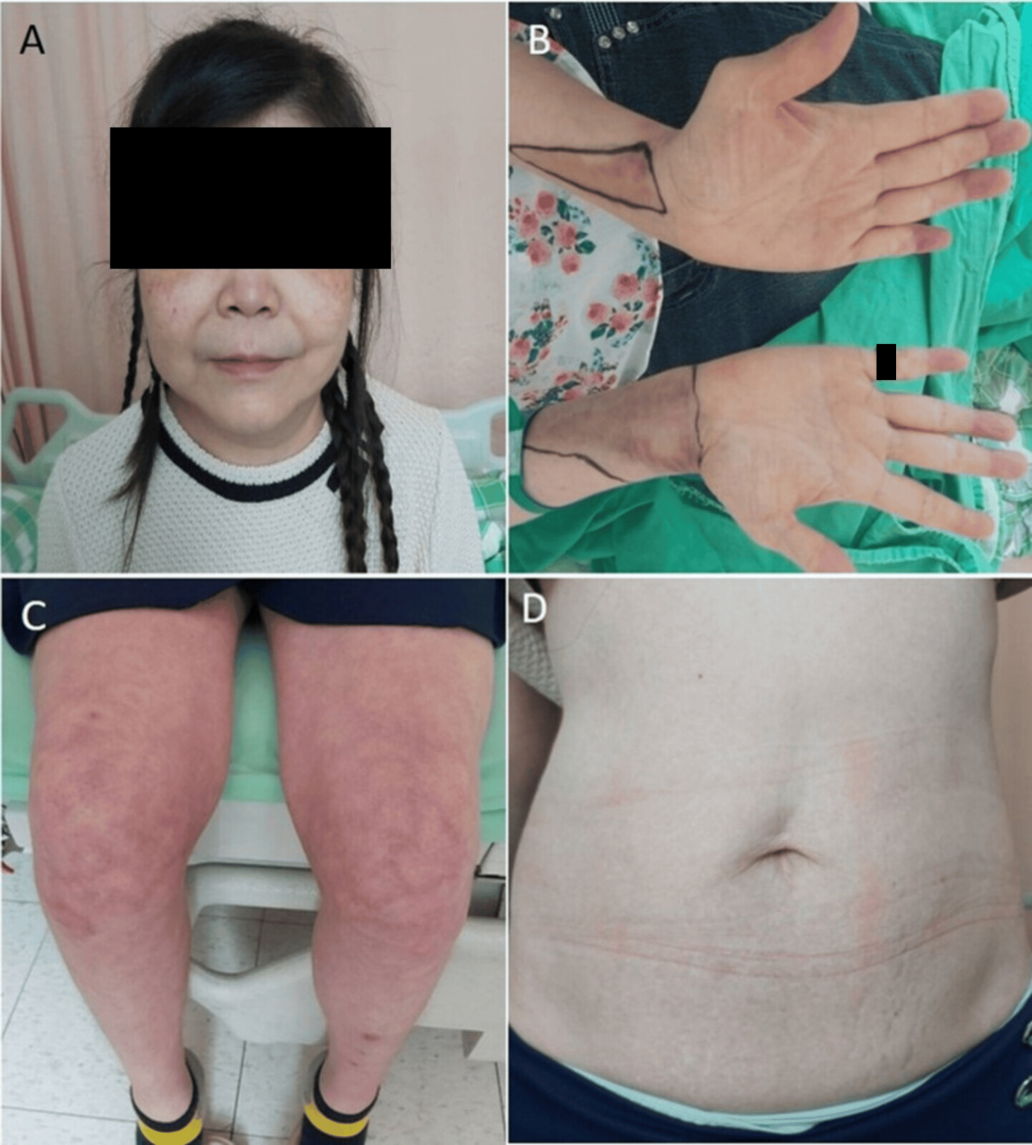
Clinical presentations on this visit (A) Facial plethora with moon face. (B) Easy bruising. (C, D) Stretch marks (striae) and abdominal striae.

A 53-year-old woman returned to the clinic with clinical features highly suggestive of CS. Her symptoms included a rounded face, reddish-purplish skin lesions over her cheeks, lower abdomen, and bilateral calves (Figure 1), along with decreasing muscle power in her bilateral proximal lower limbs, agitated mood, headache, increased hair loss, and abdominal discomfort.

The patient denied a family history of CS, but she had experienced similar symptoms 19 years ago. At that time, ACTH-independent CS was confirmed via a low-dose dexamethasone suppression test (DST) (Table 1).

**Table 1:** Endocrinological study 19 years ago *Two-day low-dose DST: dexamethasone 0.5 mg every six hours for two days and blood sampling for cortisol at 08:00 am on the third day. DST, dexamethasone suppression test

Test	Examination data	Reference range
Baseline plasma cortisol (µg/dL)		
08:00 am	31.8	5.0-19.4
05:00 pm	29.9	2.5-11.7
Baseline 24 hours urinary free cortisol (µg/24 hours)	436.2	-
Baseline plasma ACTH (µg/mL) at 08:00 am	1.4	-
Two-day low-dose DST*		
Plasma cortisol at 08:00 am	29.6	<1.8

She subsequently underwent a left-sided adrenalectomy, and the pathology report confirmed a cortical adenoma. However, the patient did not adhere to a regular post-operative follow-up schedule.

Upon presentation on this visit, physical examination revealed classic signs of hypercortisolism, including central obesity, moon face, buffalo hump, and lower abdomen striae. Her vital signs and routine laboratory data were largely within normal limits, with blood pressure at 109/71 mmHg, fasting glucose at 87 mg/dL, and HbA1c at 6.5%.

Workup was initiated to confirm the recurrence of CS (Table 2).

**Table 2:** Endocrinological study done during this visit *Two-day low-dose DST: dexamethasone 0.5 mg every six hours for two days and blood sampling for cortisol at 08:00 am on the third day. **Two-day high-dose DST: dexamethasone 2 mg every six hours for two days and blood sampling for cortisol at 08:00 am on the third day. DST, dexamethasone suppression test

Test	Examination data	Reference range
Baseline plasma cortisol (µg/dL)		
08:00 am	24.06	4.82-19.5
05:00 pm	22.84	2.47-11.9
Baseline 24 hours urinary free cortisol (µg/24 hours)	344.4	
Baseline plasma ACTH (µg/mL) at 08:00 am	2.4	
Two-day low-dose DST*		
Plasma cortisol at 08:00 am	21.22	<1.8
Two-day high-dose DST**		
Plasma cortisol at 08:00 am	22.64	-

Baseline plasma cortisol levels showed a loss of diurnal rhythmicity, and the baseline ACTH level was low at 2.4 µg/mL, reinforcing the ACTH-independent etiology. Both the standard two-day low-dose (0.5 mg/six hours) and high-dose (2 mg/six hours) DSTs failed to suppress plasma cortisol levels. Further endocrinology tests ruled out hyperaldosteronism and pheochromocytoma.

**Figure 2:**
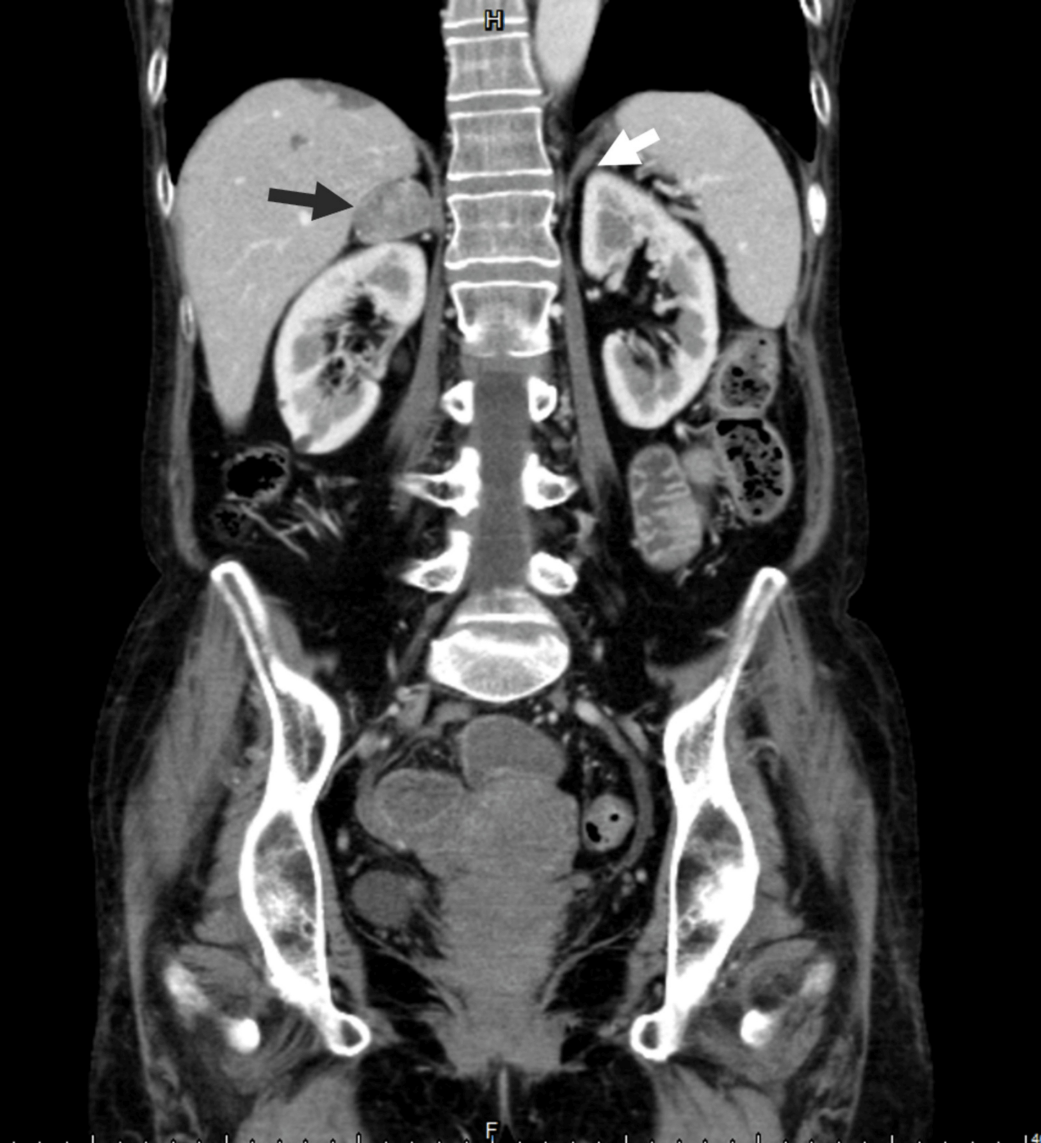
Contrast-enhanced abdominal computed tomography done during this visit (A) Black arrow, a 2.9 cm cyst-like, unenhanced nodule in the right adrenal gland. (B) White arrow, absence of the left adrenal gland due to the prior adrenalectomy.

Contrast-enhanced abdominal computed tomography (CT) confirmed the absence of the left adrenal gland due to the prior adrenalectomy but identified a 2.9 cm cyst-like, unenhanced nodule in the right adrenal gland, highly suspicious for an adrenal adenoma (Figure 2).

A pituitary gland MRI was also performed, revealing a small, poorly enhanced nodular lesion (0.3 × 0.7 × 0.4 cm) suspected to be a Rathke’s cleft cyst or microadenoma, but an ACTH-producing pituitary adenoma was effectively ruled out by the low serum ACTH level. Furthermore, a bone density examination showed osteoporosis over the lumbar region (T-score -3.8).

Given the strong suspicion of primary CS caused by the right adrenal adenoma, a laparoscopic-assisted right-sided adrenalectomy was performed. The histological examination of the resected tumor confirmed another adrenal cortical adenoma, characterized by both clear cells with abundant lipid droplets and compact, lipid-sparse eosinophilic cells, with no evidence of malignancy. Following the bilateral adrenalectomy (BADX), the patient required immediate cortisol replacement therapy. This was initiated with intravenous hydrocortisone and transitioned to oral cortisone acetate, along with oral fludrocortisone 0.1 mg per day. The maintenance regimen was established as 50 mg of cortisone acetate at 09:00 am and 25 mg at 05:00 pm. Post-operative monitoring showed that the 08:00 am baseline cortisol levels dropped significantly (3.13 µg/dL at day five and 2.29 µg/dL on one-month follow-up), indicating the successful removal of the autonomous cortisol-secreting source. The ACTH level remained low, which was suspected of being related to the steroid replacement dose.

**Figure 3:**
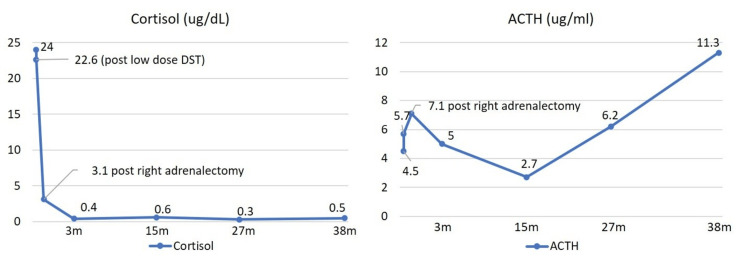
Post-operative cortisol and ACTH levels (A) Cortisol level failed to suppress under the low-dose dexamethasone suppression test, but had significantly dropped after right adrenalectomy, and remains low during follow-up. (B) ACTH level remains in the normal range with no evidence of over-production. ACTH, adrenocorticotropic hormone

Over the subsequent 38 months after the second adrenalectomy, the patient has been maintained on the established steroid replacement regimen and has experienced no symptoms of glucocorticoid deficiency or recurrent CS. Her body weight has decreased, and her round face has improved. She also received several doses of Denosumab for the management of osteoporosis. Serial brain MRI scans were conducted to monitor the risk of Nelson’s syndrome (NS), a potential complication following BADX. The small cystic pituitary lesion has remained stable in size, and the patient’s ACTH levels have not exceeded the normal upper limit, thus minimizing the risk of this syndrome at the current follow-up (Figure 3).

## Discussion

Cases of BAA causing ACTH-independent CS only occurred in 10-15% of all cases of ACTH-independent CS, which is much less than unilateral adrenal adenoma [10]. Furthermore, cases of non-simultaneous BAA are even rarer; only five cases have been reported to date. Three were independent case reports with a duration of seven to 10 years [7-9], and two cases were included in a long-term follow-up study with a duration of 20 years; all cases are listed in Table 3. Our case has the longest duration of two events that had been reported detailly, which is 19 years.

However, it remains unclear whether this presentation represents a sporadic concurrence of two independent clonal neoplasms or reflects an underlying genetic predisposition, such as germline mutations involving ARMC5, PRKACA, or GNAS. Accordingly, genetic testing may be considered in patients with BAAs, as these mutations can be associated with systemic manifestations, including meningiomas, cardiac myxomas, acromegaly, and café-au-lait macules [11].

CS may cause several consequences, such as neuropsychological changes, including depression in 86% of patients. These can persist even after successful treatment [12]. Typical dermatologic changes in CS include bruising and purple striae. Patients often experience proximal muscle wasting/weakness and osteoporosis (in 76% of patients), frequently resulting in fractures or back pain [13].

Infertility due to menstrual irregularity of 80% female patients or decreased serum concentration of sex hormones was also reported [14]. Metabolic disturbances, including hyperglycemia in 20-45% of patients, also central obesity and fat accumulation happen [15].

Most importantly, patients are at an increased risk of cardiovascular diseases such as myocardial infarction, stroke, and thromboembolism [16]. Mancini et al. had found that 80% CS patients had a higher cardiovascular risk [17].

Mortality rates for CS remain high, with an overall standardized mortality ratio of 3.0 reported by Limumpornpetch et al. [18]. The leading causes of death are atherosclerotic diseases (43.4%) and infection (12.7%) [18]. This high mortality underscores the critical need for regular follow-up and patient education on typical CS symptoms after initial adrenal surgery, as subsequent adrenal masses can arise even decades later.

Typical CS cases often presented with rapid weight gain and fat accumulation, while some were incidentalomas. Our patient, who was lost to follow-up after her first adrenalectomy, returned with typical recurrent CS features, including central obesity, buffalo hump, and lower abdomen striae. Serious consequences like osteoporosis and mood changes were also prominent [19].

The typical triad for NS includes hyperpigmentation, elevated ACTH, and progressive pituitary adenoma. However, radiology evidence of an expanding pituitary tumor is now the major diagnostic criterion, though the clinical and biochemical signs remain supporting evidence.

Papakokkinou et al. found that NS prevalence is 26% after BADX for Cushing's disease, occurring from two months to 39 years post-surgery, with prevalence reaching 38% when using MRI for follow-up [20]. Due to this high prevalence and treatment difficulty, MRI surveillance is recommended: three months post-BADX, then annually for three years, followed by every two to four years based on ACTH levels and clinical signs. Pituitary surgery or radiotherapy is the first-line treatment for NS, but prophylactic pituitary radiation before BADX is not recommended.

## Conclusions

This rare presentation of metachronous ACTH-independent CS caused by BAAs, with a remarkably long latency of 19 years between disease manifestations, underscores the need for indefinite clinical surveillance after unilateral adrenalectomy, as contralateral adrenal disease may emerge decades following initial treatment. Optimal management requires a multidisciplinary approach, encompassing precise biochemical assessment, appropriately timed surgical intervention, and comprehensive post-operative care. Additionally, continuous monitoring is essential to manage long-term risks, specifically the development of NS following BADX.
